# Interleaving Motor Sequence Training With High-Frequency Repetitive Transcranial Magnetic Stimulation Facilitates Consolidation

**DOI:** 10.1093/cercor/bhz145

**Published:** 2019-08-02

**Authors:** Jost-Julian Rumpf, Luca May, Christopher Fricke, Joseph Classen, Gesa Hartwigsen

**Affiliations:** 1 Department of Neurology, University of Leipzig, Leipzig, Germany; 2 Lise Meitner Research Group Cognition and Plasticity, Max Planck Institute for Human Cognitive and Brain Sciences, Leipzig, Germany

**Keywords:** motor consolidation, motor learning, primary motor cortex, training, transcranial magnetic stimulation

## Abstract

The acquisition of novel motor skills is a fundamental process of lifelong learning and crucial for everyday behavior. Performance gains acquired by training undergo a transition from an initially labile state to a state that is progressively robust towards interference, a phenomenon referred to as motor consolidation. Previous work has demonstrated that the primary motor cortex (M1) is a neural key region for motor consolidation. However, it remains unknown whether physiological processes underlying posttraining motor consolidation in M1 are active already during an ongoing training phase or only after completion of the training. We examined whether 10-Hz interleaved repetitive transcranial magnetic stimulation (i-rTMS) of M1 during rest periods between active motor training in an explicit motor learning task affects posttraining offline consolidation. Relative to i-rTMS to the vertex (control region), i-rTMS to the M1_hand_ area of the nondominant hand facilitated posttraining consolidation assessed 6 h after training without affecting training performance. This facilitatory effect generalized to delayed performance of the mirror-symmetric sequence with the untrained (dominant) hand. These findings indicate that posttraining consolidation can be facilitated independently from training-induced performance increments and suggest that consolidation is initiated already during offline processing in short rest periods between active training phases.

## Introduction

The acquisition of novel motor skills is fundamental for successful everyday behavior across the life-span. A large body of knowledge about motor learning is derived from motor sequence learning, a paradigm that assesses the ability to integrate different items of a movement into a coherent, effortlessly performed unit. It is generally argued that motor learning evolves across repeated practice and different phases that are believed to be sustained by distinct mechanisms (Doyon et al. 2009; Dayan and Cohen 2011; Censor et al. 2012). An initial learning phase during which performance improves within session as a function of practice (referred to as fast online learning) is followed by an offline phase during which the initially labile motor memory is transformed into a more robust representation in the absence of further practice, a phenomenon referred to as motor consolidation (e.g., Karni et al. 1998; Muellbacher et al. 2002; Doyon et al. 2003; Krakauer et al. 2005; Robertson et al. 2005; Hadipour-Niktarash et al. 2007). In recent years, a large number of studies explored the potential of noninvasive brain stimulation (NIBS) techniques to modulate posttraining offline consolidation of motor learning. Overall, these previous studies demonstrated a fundamental role of the primary motor cortex (M1) for offline processing of training-induced performance increments after termination of active training (see Dayan and Cohen 2011 for a review). Accordingly, inhibition of M1 with immediate posttraining low-frequency repetitive transcranial magnetic stimulation (rTMS) was demonstrated to disrupt consolidation of motor skills (Muellbacher et al. 2002; Robertson et al. 2005). Enhancing M1 excitability directly after training by use of remote application of theta-burst stimulation, on the other hand, induced offline performance increments during consolidation (Tunovic et al. 2014). Other studies have demonstrated that anodal transcranial direct current stimulation (tDCS) of M1 may improve offline motor consolidation when applied after training (Tecchio et al. 2010; Rumpf et al. 2017, 2018) or concurrently with ongoing motor training (Reis et al. 2009, 2015). Notably, tDCS was applied for a duration of 20 min during training in the latter studies. Stimulation durations of 3 min or longer, however, were shown to induce aftereffects that persist for minutes up to hours depending on stimulation duration (Nitsche and Paulus, 2000, 2001). Therefore, effects on posttraining consolidation induced by the application of tDCS concurrently with ongoing motor training cannot be dissociated from effects induced by an interaction of tDCS aftereffects with consolidation in these studies. Moreover, motor sequence learning paradigms usually employ repeated blocks of active training, which are interspersed with short rest periods to prevent fatigue. Bonstrup et al. (2019) recently demonstrated that the training-acquired motor engram is consolidated already during these short rest periods (hereafter referred to as short-term offline processing). Consequently, continuous application of NIBS during a motor training session (or before, in case of persisting aftereffects) likely interacts with both online processing in M1 and short-term offline processing between active training blocks. Therefore, this approach does not allow differentiating NIBS effects on online motor learning from effects on short-term offline processing during an ongoing training session, and it remains unknown how this affects offline consolidation after termination of training. A better understanding of these processes would be of great interest to deepen the current knowledge on the role of M1 in motor consolidation and increase the efficiency of NIBS applications.

Here, we aimed at directly modulating offline processing in M1 between active learning blocks without affecting online training performance. We hypothesized that interventions during rest blocks following active training blocks may have effects on motor consolidation that could be dissociated from effects on active motor training. Consequently, we applied short bursts of interleaved rTMS (i-rTMS) directly after each training block. To this end, we used trains of 10-Hz rTMS, which has been demonstrated to transiently modulate cognitive processing across a variety of domains (e.g., attention, action reprogramming, language, and mental imagery) when applied during task execution (e.g., Rushworth et al. 2001; Devlin et al. 2003; Gough et al. 2005; Sack et al. 2005; Hartwigsen et al. 2010, 2012). The interleaved application of short rTMS bursts allowed us to modulate offline processing without direct interference with active motor training. We expected that our interleaved approach would interact with offline processing of motor representation formation between active training blocks without affecting training performance per se.

Notably, the direction of the modulatory effects of rTMS in studies relying on behavioral measurements (such as speed or task accuracy) is not clear to date (e.g., Sliwinska et al. 2017) and seems to crucially depend, among other factors, on the current brain state (Silvanto et al. 2008; Silvanto and Cattaneo 2017) and timing of the rTMS application with respect to task execution (Luber et al. 2007). Indeed, several studies report facilitation or enhancement rather than deterioration of behavior during or after high-frequency rTMS in different cognitive tasks (Kohler et al. 2004; Mottaghy et al. 2006; Luber et al. 2007; Preston et al. 2010; Andoh and Paus 2011; Andoh and Zatorre 2013). Moreover, motor cortex excitability is usually increased when rTMS is given at frequencies higher than 1 Hz (Pascual-Leone et al. 1994; Jennum et al. 1995; Chen et al. 1997; Berardelli et al. 1999). Consequently, i-rTMS might either disrupt or facilitate offline consolidation of motor skills.

We found that i-rTMS of M1 during a motor sequence learning session selectively facilitated posttraining offline motor consolidation assessed several hours after training without affecting online learning. Initiation and malleability of posttraining consolidation already during short pauses between active training blocks may have implications for the mechanisms underlying motor consolidation and stimulate novel therapeutic strategies in motor rehabilitation.

## Materials and Methods

### Participants

Twenty-four right-handed healthy individuals aged between 21 and 30 years (mean age = 27.4 years; 12 females) who were naïve to the motor sequence learning task and the purpose of the experiment were recruited from a participant database at the Max Planck Institute for Human Cognitive and Brain Sciences. All participants were right-handed according to the German version of the Edinburgh Handedness Inventory (Oldfield 1971) (mean laterality quotient = 92.7, standard deviation [SD] 8.3). None of the participants reported a history of neurological, psychiatric or other serious medical diseases, and none were professional musicians or had been trained as typists. Using the Beck Depression Inventory (BDI) (Beck et al. 1974), potential participants were screened for symptoms of depression and were excluded from participation in the study, if the BDI score was >19 (mean BDI score = 2.8, SD 3.6). Additional exclusion criteria encompassed central nervous system active medication and contraindications against TMS. The study protocol conformed to the principles of the Declaration of Helsinki and was approved by the local ethics committee at the University of Leipzig. All participants gave their written informed consent to participate in the study.

### Experimental Procedure

All participants took part in 2 experimental sessions corresponding to 1 of 2 different i-rTMS sites: i-rTMS directed to the hand M1 region in the right hemisphere (M1_hand_) and i-rTMS directed to the vertex. Each session encompassed 2 phases: a motor sequence training part in the morning (between 9 and 12 AM) during which the i-rTMS intervention was applied and a delayed retest after 6 h to assess consolidation of training-acquired task skill. The order of sessions was counterbalanced across subjects, and both sessions were separated by at least 14 days. Prior to each training and retest session, alertness was assessed with the Stanford Sleepiness Scale (Hoddes et al. 1973).

**Figure 1:**
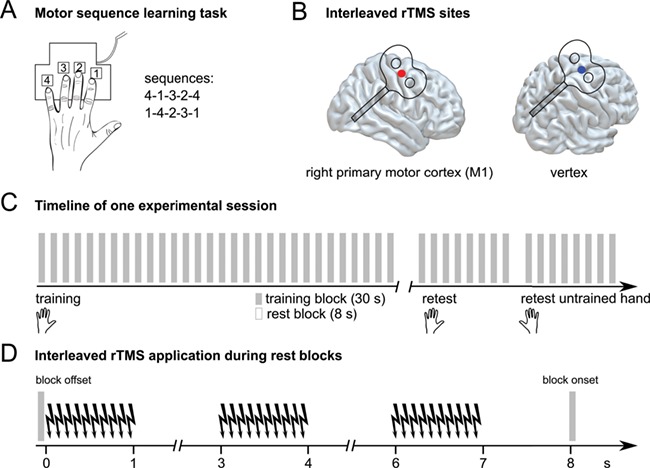
Experimental design. The experiment consisted of 2 sessions that were performed on separate days (intersession interval > 14 days). (*A*) Participants performed a different 5-item motor sequence in each session with their left hand. (*B*) i-rTMS was applied over either the right M1_hand_ area or vertex in different sessions. (*C*) During each session, participants performed 30 training blocks of an explicit motor sequence learning task with their left hand, interleaved by short rest blocks. Offline consolidation of training-induced performance increments was assessed 6 h later with the trained hand, immediately followed by retesting the mirror-symmetric sequence with the untrained hand. (*D*) Three trains of i-rTMS were applied during the 8-s rest blocks, starting at 0 s, 3 s, and 6 s (jitter: ±50 ms) after the offset of the last sequence of the active training block.

### Motor Sequence Learning Task

Motor sequence learning was assessed by an adapted version of the explicit sequential finger-tapping task introduced by Karni et al. (1995). In each experimental session, participants were asked to practice 1 of 2 different, equally difficult 5-item finger-tapping sequences with their left (nondominant) hand: sequence 1, “4-1-3-2-4”; and sequence 2, “1-4-2-3-1” (1 = index finger, 2 = middle finger, 3 = ring finger, and 4 = little finger). Sequence 1 and sequence 2 were counterbalanced across subjects with respect to assignment to the 2 different TMS sessions. To verify explicit knowledge of the sequence prior to the training phase, participants were required to slowly repeat the finger movement sequence until they managed to correctly reproduce it 3 times in a row. The following task training phase encompassed 30 successive blocks of task execution separated by 8-s rest blocks during each of which TMS was applied to M1_hand_ or vertex as described below (cf. Fig. 1). During training, participants were instructed to perform the sequence as rapidly as possible while making as few errors as possible. No information about the sequence was given during task training. Unbeknownst to the participants, each practice block was automatically terminated after 30 key presses to control for the number of movements. Hence, a maximum of 6 correct sequence repetitions could be executed within one single block of training. Participants were instructed to continue practicing by again starting at the beginning of the sequence if they became aware that they had made an error. The beginning of a training block was indicated by a green fixation cross in the middle of a computer screen in front of the participants, which changed to red color (after 30 key presses) to indicate the beginning of a rest block. During rest blocks, participants were instructed to relax their hand until the start of the next practice block. The delayed retest session after an interval of 6 h consisted of 8 blocks of the task. Afterwards, participants were asked to also perform the retest with their right (dominant) hand. Motor sequence performance recording was employed using customized MATLAB scripts (MathWorks).

### Interleaved rTMS

We used frameless stereotaxy (Localite TMS Navigator) based on the coregistered individual *T*_1_-weighted magnetic resonance image to navigate the TMS coil and maintain the exact location and orientation throughout the experimental sessions. As a prerequisite for stereotactic coil placement, *T*_1_-weighted images were taken from the in-house database at the Max Planck Institute for Human Cognitive and Brain Sciences. Each experimental session started with individual coregistration. Thereafter, the individual resting motor threshold (RMT) was determined. RMT was defined as the lowest intensity that caused 5 out of 10 motor evoked potentials (MEPs) with a size of at least 50 μV in the relaxed first dorsal interosseous muscle of the left hand when stimulating the hand region of right M1 with single-pulse TMS. The physiologically defined individual motor hotspot (M1_hand_) was marked in the neuronavigation software and used for coil placement in the main experiment. Thereafter, neuronavigated rTMS was applied over either right M1_hand_ or vertex in 2 different sessions (Fig. 1*B*). We chose the vertex as the control site because this area is not associated with hand movements and has been previously introduced as a valid control condition in different motor studies (Bestmann et al. 2002; Grefkes et al. 2010; Jung et al. 2016). The vertex was defined as Cz by the 10–20 EEG system. rTMS was given in an interleaved fashion in each of the 8-s rest blocks between training blocks. To this end, 3 bursts of 10 pulses at 10 Hz were applied at 0, 3, and 6 s after training offset. Burst onsets were jittered with an interval of ±50 ms at 3 and 6 s to avoid habituation and reduce predictability of i-rTMS application (Fig. 1*D*).

Stimulation intensity for i-rTMS was set to 90% of the RMT. Stimulation at this intensity has been associated with task interference in our previous studies targeting various regions with rTMS during task processing (Hartwigsen et al. 2010, 2012, 2016). The coil was oriented 45° to the sagittal plane, such that the second phase of each biphasic pulse induced a posterior-to-anterior current flow in the brain. A MagPro X100 stimulator (MagVenture 4.3.20, Medtronic) equipped with a focal figure-of-eight coil (MC-B70; outer diameter = 9.7 cm) was used for TMS application. Prior to TMS application, participants were equipped with earplugs to shield them from the TMS-induced noise. The overall application of TMS pulses per session was well within the recommended safety limits (see Rossi et al. 2009, Rossini et al 2015).

### Data Analysis

Data were processed using customized MATLAB scripts (MathWorks) to extract speed performance and accuracy. Speed was defined as the average time (in seconds) needed to complete correct sequences within each block (time to complete correct sequences, TCS). Accuracy was defined as the number of correct sequences in a given block. As speed and accuracy were equally important components of the motor sequence learning task (i.e., participants were instructed to perform the sequence as fast as possible while making as few errors as possible) and to account for interindividual differences with respect to the strategy to improve task performance (e.g., prioritize speed performance at the expense of accuracy), performance was assessed with a variable performance index (PI; Dan et al. 2015; King et al. 2017) that incorporates speed performance and accuracy according to the following formula (larger PI values indicate better task performance):

$$\begin{equation*} \textrm{PI}_{\textrm{x}} = \textrm{exp}^{-(\textrm{TCS}_{\text{x}})}\ {}^{\ast}\ \textrm{exp}^{-(\textrm{Errors}_{\text{x}}/6)}\ {}^{\ast}\ 100 \end{equation*}$$

where x = task block and Errors = maximum number of correct sequences (i.e., 6) minus the number of actual correct sequences within each block.

The effect of the different i-rTMS interventions on performance development during the training phase was assessed using a repeated measures analysis of variance (rmANOVA) with block (30 levels) and i-rTMS site (2 levels: M1_hand_ or vertex) as within-subject factors. This allowed us to test for performance changes as a function of training (main effect of block), for differences in the rate of learning with respect to the site of i-rTMS intervention during the training phase (block × TMS site interaction), and for overall task performance differences during training between the 2 i-rTMS sites (main effect of i-rTMS site). “End-of-training performance” was examined separately using another rmANOVA with block (4 levels: last 4 blocks of training) and i-rTMS site (2 levels) as within-subject factors to evaluate whether participants reached asymptotic task performance (main effect of block) at the end of the training phase in both stimulation sessions (block × i-rTMS site interaction).

To quantify consolidation, offline changes of PI measures were assessed between the individual end-of-training performance (EOT; i.e., average PI of the last 4 blocks of training) and each block of delayed retesting according to the following formula:

$$\begin{align*} \textrm{normalized PI}_{\textrm{x}} =&\ (\textrm{retest}\_\textrm{PI}_{\textrm{x}} - \textrm{PI}_{\textrm{EOT}})/\textrm{PI}_{\textrm{EOT}},\\ &\qquad\textrm{where}\ \textrm{x} = \textrm{retest block}. \end{align*}$$

Therefore, normalized PI values (reported as percentage) above zero indicate gains of task performance relative to the individual end-of-training performance, while negative normalized PI values indicate performance decrements relative to end-of-training performance.

An rmANOVA of these normalized PI measures with block (8 levels) and i-rTMS site (2 levels) as within-subject factors allowed us to test for overall differences of consolidation between both stimulation sessions (main effect of i-rTMS site). This procedure also provided information on performance changes driven by additional task training during the retest (main effect of block) and on potential differences between both i-rTMS sites with respect to learning rate during the retest (block × i-rTMS site interaction).

One participant was excluded from the analysis due to decreasing performance (i.e., “unlearning” the task) across blocks of training in both sessions, which resulted in lower end-of-training performance than in the initial block. Hence, data of 23 participants were entered in the final analysis.

For all statistical tests, the alpha level was set to *P* < 0.05. In case of violation of the sphericity assumption, Greenhouse–Geisser corrections were applied. PI measures are reported as mean with 95% confidence interval (CI). All statistical analyses were conducted with SPSS 24 (SPSS).

**Figure 2:**
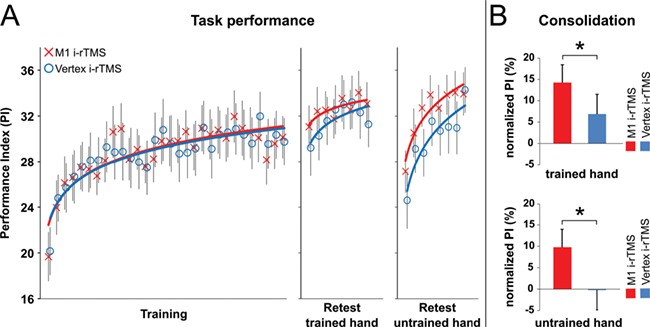
Behavioral results. (*A*) Task performance. PI measures across blocks of training (30 blocks), delayed retesting of the trained (left) hand (8 blocks), and delayed retesting of the untrained (right) hand (8 blocks). Vertical bars represent standard error of the mean (SEM). (*B*) Consolidation. Columns represent the mean of normalized PI measures across the 8 blocks of delayed retesting, that is, retest PI changes relative to the individual end-of-training performance of the trained hand (average PI of the last 4 blocks of training). Bars represent SEM. The asterisk (*) indicates significant difference of consolidation following i-rTMS directed to M1 relative to i-rTMS directed to vertex (*P* < 0.05).

## Results

### i-rTMS During Motor Training Does Not Affect Training-Induced Performance Changes

A rmANOVA conducted on the PI values of the 30 blocks of training during both sessions (i-rTMS directed to the M1_hand_ area and i-rTMS directed to the vertex) revealed a significant main effect of block (*F*_29,638_ = 11.249, *P* < 0.001), with no significant effect of i-rTMS site (*F*_1,22_ = 0.002, *P* = 0.968), nor a significant block × i-rTMS site interaction (*F*_29,638_ = 0.704, *P* = 0.876). Initial training PI (first block) was 19.7 (95% CI 15.1–24.2) in the M1_hand_ i-rTMS session and 20.1 (95% CI 15.7–24.6) in the vertex i-rTMS session and increased to 30.1 (95% CI 26.2–34.1, M1_hand_ i-rTMS) and 29.7 (95% CI 25.4–34.1, vertex i-rTMS) in the last block of training. These results indicate that, while all participants improved task performance across training in both sessions, neither overall task performance nor the rate of learning was modulated by M1_hand_ or vertex i-rTMS (Fig. 2*A*).

Another rmANOVA performed on the PI values of the last 4 blocks of training revealed no significant main effect of block (*F*_3,66_ = 1.716, *P* = 0.172) and no significant main effect of i-rTMS site (*F*_1,22_ = 0.759, *P* = 0.393), nor a significant block × i-rTMS site interaction (*F*_3,66_ = 0.753, *P* = 0.525). End-of-training performance (i.e., average PI of the last 4 blocks of training) amounted to 29.5 (95% CI 25.4–33.6) during training in the M1_hand_ i-rTMS session and reached 30.4 (95% CI 26.1–34.7; *P* = 0.393) when i-rTMS during training was directed to the vertex. This indicates that block-to-block performance changes leveled off by the end of the training and verifies that participants reached a similar asymptotic end-of-training performance level in both sessions (Fig. 2*A*).

### i-rTMS of M1 During Training Facilitates Motor Consolidation

A rmANOVA conducted on the normalized PI values of the 8 retest blocks demonstrated a weak trend for the main effect of block (*F*_2.878,63.324_ = 2.302, *P* = 0.088) but no significant block × i-rTMS site interaction (*F*_4.176,91.871_ = 0.741, *P* = 0.572), suggesting that additional performance increments across blocks of delayed retesting (online learning) were comparable in both the M1_hand_ and vertex i-rTMS sessions. To minimize contamination of offline performance changes with additional practice (“online learning”) on the task, consolidation was first assessed between the initial block of the retest session and the end-of-training performance. A rmANOVA on the normalized PI of the first block of delayed retesting in both sessions revealed a significant main effect of i-rTMS site (*F*_1,22_ = 6.842, *P* = 0.016; partial η^2^ = 0.237), which was driven by offline increments of initial retest performance in the M1_hand_ i-rTMS session (PI of the first retest block in relation to end-of-training performance: +8.3%, 95% CI −0.7 to 17.3), while initial retest performance in the vertex i-rTMS session showed a decrement compared with the individual end-of-training performance level (−4.0%, 95% CI −13.6 to 5.7). As performance is typically variable and to exclude that results were confounded by “warming-up” effects that may be observed at the beginning of delayed retesting, we additionally assessed consolidation based on the average normalized performance across all 8 blocks of delayed retesting, which did not affect the overall pattern of results (main effect of i-rTMS site: *F*_1,22_ = 5.389, *P* = 0.030; partial η^2^ = 0.197). These findings were driven by improved consolidation in the M1_hand_ i-rTMS session (average normalized retest PI: +14.3%, 95% CI 5.4–23.1) compared with vertex i-rTMS (+6.9%, 95% CI −2.9 to 16.7; Fig. 2*B*). Noteworthy, the fact that the 95% CI of average normalized retest PI included zero (i.e., the end-of-training performance) in the vertex i-rTMS session, but excluded zero in the M1_hand_ i-rTMS session, indicates that only i-rTMS of M1_hand_—besides facilitating consolidation relative to vertex i-rTMS—induced substantial delayed retest performance improvement compared with the individual end-of-training performance.

Taken together, the above results point to a beneficial effect of i-rTMS over M1 during finger sequence training with respect to posttraining consolidation processes.

### Improved Consolidation Induced by i-rTMS of M1 Generalizes to the Untrained Hand

To assess potential effects of i-rTMS on delayed retest performance of the untrained hand, we applied an rmANOVA with the within-subject factors block (8 levels) and i-rTMS site (2 levels) on the PI values of the 8 retest blocks performed with the untrained right hand (following left-hand retesting). Due to missing data of the right-hand retest of 2 participants, this analysis could be performed for only 21 participants. Results revealed a significant main effect of block (*F*_7,140_ = 12.399, *P* < 0.001) but no significant block × i-rTMS site interaction (*F*_7,154_ = 0.741, *P* = 0.572), suggesting similar online performance increments across retesting for both i-rTMS sessions. More importantly, the results demonstrated a significant main effect of i-rTMS site (*F*_1,20_ = 5.665, *P* = 0.027; partial η^2^ = 0.221), which was driven by larger retest PI values across retesting in the M1_hand_ i-rTMS session (average retest PI: 32.3, 95% CI 27.9–36.8) compared with retest performance of the untrained hand in the vertex i-rTMS session (average retest PI: 30.1, 95% CI 26.1–34.2; Fig. 2*A*). This indicates not only that i-rTMS of M1_hand_ during training improved delayed retest performance of the trained hand but that this effect also generalized to the untrained hand.

In an additional exploratory analysis and to be consistent with the consolidation measure of the trained hand, retest PI values of the untrained hand were normalized to the individual end-of-training performance of the trained hand. A rmANOVA applied to these normalized retest PI values of the untrained hand demonstrated a significant main effect of i-rTMS site (*F*_1,20_ = 7.868, *P* = 0.011; partial η^2^ = 0.282) and block (*F*_7,140_ = 11.486, *P* < 0.001) in the absence of a significant block × i-rTMS site interaction (*F*_4.451,89.029_ = 0.730, *P* = 0.588). The significant main effect of block was driven by improved normalized retest performance of the untrained hand in the M1_hand_ i-rTMS session (average normalized retest PI: +9.8%, 95% CI 2.8–16.1) compared with the vertex i-rTMS session (−0.2%, 95% CI −8.1 to 7.6), which further supports the finding that beneficial effects on consolidation induced by i-rTMS of M1_hand_ during training generalized to the untrained hand (Fig. 2*B*).

Subjective alertness as assessed by the Stanford Sleepiness Scale was not significantly different between the 2 i-rTMS conditions during training or retest (all *P*s > 0.05).

### i-rTMS Does Not Induce Aftereffects on Corticospinal Excitability

Finally, we conducted a control experiment to test for potential aftereffects of i-rTMS on corticospinal excitability that may have persisted into the training blocks. To this end, we included a new sample of 12 healthy right-handed subjects (6 females, age range: 27–37 years; mean age = 29.25 years, SD 4.07; mean laterality index = 99.08%, SD 3.89 [Oldfield 1971]). The experimental design was similar to the main experiment but included a single MEP measurement during each “training” block instead of active motor training. First, RMT and 1-mV threshold were determined, and 20 baseline MEPs were recorded at an intensity of the 1-mV threshold with the TMS coil placed over the right M1_hand_ area. Matching the motor learning experiment, we then alternated 30 MEP blocks with 30 “rest” blocks with i-rTMS intervention. During each MEP block, which lasted for 8 s, a single MEP was recorded 2 s after offset of the preceding i-rTMS train. TMS pulses for evoking the MEP were delivered at the intensity of the 1-mV threshold. During the alternating 8-s “rest” blocks, 3 trains of 10-Hz i-rTMS with 10 pulses each were applied to the right M1_hand_ area at 0, 3, and 6 s after MEP block offset at an intensity of 90% RMT. Stereotactic neuronavigation was used to maintain a stable coil position across the experiment.

For each subject, MEPs from the 30 MEP blocks were normalized to the averaged individual baseline MEPs (MEP_baseline_) according to the following formula:

$$\begin{align*} \textrm{normalized MEP}=&\ ({\textrm{MEP}_{\textrm{x}}}-{\textrm{MEP}_{\textrm{baseline}}})/ {\textrm{MEP}_{\textrm{baseline}}},\\ &\qquad\textrm{where x}=\textrm{MEP block}.\end{align*}$$

A one-sample *t*-test of the individual averages of normalized MEPs was then used at the group level to test whether i-rTMS modulated corticospinal excitability when probed 2 s after the offset of the i-rTMS train. Results revealed a nonsignificant decrease of the mean MEP amplitude relative to baseline after i-rTMS (−6.8%, 95% CI −20.1% to 6.5%; *t*_11_ = −1.13, *P* = 0.283). In summary, we did not find evidence for aftereffects of i-rTMS on corticospinal excitability persisting into the training block.

## Discussion

The present study shows that application of i-rTMS targeting M1 during short pause intervals between blocks of active training facilitates posttraining offline consolidation without affecting online skill acquisition during an ongoing training session. Moreover, the facilitatory effect of our i-rTMS approach on posttraining offline consolidation also led to improved cross-limb transfer to the untrained hand at delayed retesting. Importantly, the fact that we found similar online learning during i-rTMS of M1_hand_ and i-rTMS of vertex rules out the possibility that posttraining offline consolidation was confounded by different training-induced performance increments during online learning. Rather, our results indicate that i-rTMS selectively facilitated transformation of training-induced motor skill into a robust representation by repeated interaction with short-term offline processing in M1.

Previous studies have shown that consolidation was facilitated when anodal tDCS directed to M1 (Tecchio et al. 2010; Rumpf et al. 2017, 2018) or continuous theta-burst stimulation remote from M1 (Tunovic et al. 2014) was applied immediately after termination of a motor training session. These results suggest that mechanisms underlying consolidation may not require output neurons in M1 to be active. Improved posttraining offline motor consolidation was also observed when anodal tDCS of M1 was applied concurrently with motor training (Reis et al. 2009, 2015). Note that, in these latter studies, stimulation was applied continuously across the training sessions, encompassing blocks of active training movements interleaved with rest blocks. Consequently, it was not possible to differentiate whether NIBS during training facilitated posttraining offline consolidation by an interaction with online or offline processing of motor learning. Our results indicate that posttraining consolidation indeed can be facilitated by an interaction of NIBS with short-term offline processing in M1. One open question is whether motor consolidation already started during training or whether our i-rTMS protocol induced effects that operated after completion of the training. Given that the effects of rTMS bursts are usually short-lasting (Rotenberg et al. 2014), its physiological effects are unlikely to extend beyond the end of the training. The implication is that consolidation is already initiated within short rest periods between active training blocks during an ongoing motor training session. This conclusion is supported by recent findings of Bonstrup et al. (2019) who demonstrated that performance increments during the early training phase, in which performance improves substantially across blocks, are indeed generated offline during short rest periods between training blocks, while performance did not improve online during active training. It is most likely that i-rTMS facilitated motor engram processing during these short training pauses such that these engrams could be more easily reactivated afterwards, which was reflected by improved posttraining consolidation in our study. These results raise the interesting possibility that consolidation may even be specifically tied to a brain state where output neurons of M1 remain inactive.

Tunovic et al. (2014) reported that offline performance increments during consolidation could be induced by preventing the posttraining decrease of corticospinal excitability associated with explicit motor sequence learning by use of remote theta-burst stimulation. As high-frequency (5–20 Hz) rTMS has been demonstrated to increase corticospinal excitability during trains of stimulation (Pascual-Leone et al. 1994), application of short trains of 10-Hz rTMS to M1 during the short rest periods between active training blocks, as applied in the current study, may have facilitated the induction of consolidation through a similar mechanism. However, training-induced corticospinal excitability changes during interleaving short resting periods and after training were not assessed in our study to avoid interference with task processing.

Of note, i-rTMS of M1_hand_ facilitated not only skill consolidation of the trained left hand in our study but also cross-limb transfer of skill consolidation to the untrained right hand. Several previous studies have shown transfer effects to the nontrained hand after motor sequence learning using serial reaction time tasks (e.g., Grafton et al. 2002; Perez et al. 2007, 2008). Such generalization processes engage plastic network interactions between M1 and premotor areas as well as higher-order regions in the frontal cortex (Censor and Sagi 2009; Censor et al. 2012). These effector-independent effects suggest that stimulation-induced effects may not have been restricted to the targeted right M1_hand_ but additionally induced effects in a larger neural network. A recent study demonstrated that storage of sequence-specific information relies on training-induced formation of increasingly specialized neuronal circuits, which are distributed widely across execution-related cortical areas such as M1 and also secondary cortical motor areas (Wiestler and Diedrichsen 2013). The fact that improved posttraining offline skill increments of the trained hand were transferred to the untrained hand suggests that i-rTMS interacts with the formation of such specialized neuronal circuits encoding sequence-specific information rather than unspecifically modulating motor output of the stimulated M1.

At first glance, our results appear to be in discordance with a number of previous studies that found impaired performance when 10-Hz rTMS was applied over neural key regions for different cognitive tasks (e.g., Rushworth et al. 2001; Devlin et al. 2003; Gough et al. 2005; Sack et al. 2005; Hartwigsen et al. 2010, 2012). Notably, these studies selectively assessed the immediate online impact of rTMS at the behavioral level. The discrepancy in the direction of the rTMS effect between the present and previous studies might be explained by differences in the timing of the stimulation (offline vs. online) and the affected processes. The absence of an i-rTMS effect on online learning across the training session in the present study suggests that even if immediate aftereffects of i-rTMS on the following active training blocks exist, then they seem to be too weak to actively modulate task performance.

We are not aware of any previous study using a similar design. However, Kim et al. (2004) applied 10-Hz rTMS immediately before blocks of sequential motor learning and reported improved online learning, which they attributed to rTMS-induced enhancement of training-induced plasticity in the motor cortex. Other studies applied 10-Hz rTMS bursts immediately before task processing in the language domain (e.g., Topper et al. 1998; Mottaghy et al. 1999; Sparing et al. 2001; Andoh et al. 2006; Mottaghy et al. 2006; Andoh and Paus 2011) and reported a facilitation of different language tasks. The observed facilitation of consolidation in our study might thus be explained in terms of the “state dependency” hypothesis, arguing that the (task-induced) neural brain state can modulate the impact of TMS on behavior in a qualitatively different manner, resulting in either inhibition or facilitation (Silvanto et al. 2008). Depending on the neuron population that will be activated, the TMS-induced activity can be considered both as noise and as part of the task signal (Miniussi et al. 2010). The induced activity might be synchronized with the ongoing relevant signal, thereby rendering the signal stronger and providing an “optimum” level of noise for a specific task or process (Miniussi et al. 2013), for instance, offline storage of sequence-specific information. Notably, the interaction of the current brain state, stimulation intensity, and time point of stimulation has been shown to affect the direction of the behavioral outcome of an rTMS intervention (Silvanto et al. 2017; Silvanto and Cattaneo 2017; Silvanto et al. 2018). Based on findings from the perceptual domain, Silvanto and Cattaneo (2017) proposed a nonlinear transition from impairment to facilitation with decreasing stimulation intensity. Crucially, in this framework, changes in the brain state result in a shift of the observed behavioral rTMS pattern such that intensities, which normally impair perception, can have a facilitatory effect if the initial brain state has changed. Indeed, a number of studies showed selective facilitatory rTMS effects on near-threshold stimuli (e.g., Abrahamyan et al. 2011; Schwarzkopf et al. 2011). It is reasonable to assume that, in our study, the preactivation of neurons in the motor cortex induced by the active training blocks prior to each rest period changed the excitability of these neurons, which might have turned a disruptive effect (as observed in some of the previous online 10-Hz rTMS studies cited above) into facilitation. Hence, we might speculate that i-rTMS increased activity in the targeted right motor cortex to a level that was optimal for motor consolidation and thus directly interacted with ongoing rapid consolidation processes during training breaks that later manifested in better task performance during the retest.

However, we cannot fully exclude that aftereffects of i-rTMS persisted into the following training block and may have interacted with online processing during motor sequence execution. Our i-rTMS protocol (10 pulses at 10 Hz applied at 0, 3, and 6 s after training offset) was devised as a compromise between maximizing the modulatory rTMS effect during rest and minimizing potential spillover into the next active training block. The rationale for the timing of the last onset was based on previous work, arguing that high-frequency rTMS bursts (>5 Hz) typically lead to a modulation of cortical activity at the stimulation site for a period outlasting the stimulation for about half the duration of the stimulation train (Guse et al. 2010; Rotenberg et al. 2014). Consequently, we reasoned that each of our subthreshold 10-Hz bursts should maximally influence the stimulated area for 1.5 s. The last burst was applied 6 s after training offset. As each rest block lasted for 8 s, aftereffects should be restricted to the rest block and were unlikely to spill over to the following training block. The above considerations are supported by the results of our neurophysiological control experiment, which demonstrated that i-rTMS did not induce aftereffects on corticospinal excitability that persisted into the following training block. Nevertheless, we wish to emphasize that we cannot fully exclude the possibility that aftereffects induced by i-rTMS interacted with online processing during subsequent task execution, even in the absence of any measurable immediate behavioral changes during online learning or any measurable immediate effect on corticospinal excitability at the time window during which motor sequences were executed. Such aftereffects might have enhanced learning-related synaptic connections during task performance, which might have enhanced stability of these connections and thus facilitated motor consolidation.

In conclusion, our results demonstrate that processes leading to posttraining consolidation of training-induced skill increments are already initiated during short resting periods between task execution and may be facilitated by an interaction of i-rTMS with M1 as a node of a network involved in controlling sequential finger movements. i-rTMS seems to facilitate the storage of sequence-specific information that enables improved posttraining offline processing and transfer of improved skill consolidation to the untrained hand. Importantly, since the acquisition of almost all motor skills engages motor sequence learning, the investigation of the underlying processes with a simple motor sequence learning task represents an ecologically valid paradigm for motor behavior in everyday life. Our key finding that motor consolidation can be facilitated when NIBS is applied interleaved with active training may have practical implications for therapeutic strategies, suggesting that even a single session of training combined with i-rTMS may support relearning of motor skills in patients with brain damage.

## Notes

We would like to thank Ina Koch, Laura Nieberlein, Dana Richter, and Lisa Kunz for support with the data acquisition. *Conflict of Interest:* None declared.
